# Analysis of fibrocalcific aortic valve stenosis: computational pre-and-post TAVR haemodynamics behaviours

**DOI:** 10.1098/rsos.230905

**Published:** 2024-02-21

**Authors:** Adi Morany, Ricardo Gomez Bardon, Karin Lavon, Ashraf Hamdan, Danny Bluestein, Rami Haj-Ali

**Affiliations:** ^1^ School of Mechanical Engineering, Tel Aviv University, Tel Aviv, Israel; ^2^ Dassault Systemes España, Madrid, Spain; ^3^ Department of Cardiology, Rabin Medical Center, Petach Tikva, Faculty of Medicine, Tel Aviv University, Tel Aviv 6997801, Israel; ^4^ Department of Biomedical Engineering, Stony Brook University, Stony Brook, NY, USA

**Keywords:** lattice Boltzmann method, calcific aortic valve, fluid–structure interaction, finite element, fibrosis

## Abstract

Fibro-calcific aortic valve (AV) diseases are characterized by calcium growth or accumulation of fibrosis in the AV tissues. Fibrocalcific aortic stenosis (FAS) rises specifically in females, like calcification-induced aortic stenosis (CAS), may eventually necessitate valve replacement. Fluid-structure-interaction (FSI) computational models for severe CAS and FAS patients were developed using lattice Boltzmann method and multi-scale finite elements (FE). Three parametric AV models were introduced: pathology-free of non-calcified tri-and-bicuspid AVs with healthy collagen fibre network (CFN), a FAS model incorporated a thickened CFN with embedded small calcification volumes, and a CAS model employs healthy CFN with embedded high calcification volumes. The results indicate that the interaction between calcium deposits, adjacent tissue and fibres crucially influences haemodynamics and structural reactions. A fourth model of transcatheter aortic valve replacement (TAVR) post-procedure outcomes was created to study both CAS and FAS. TAVR-CAS had a higher maximum contact pressure and lower anchoring area than TAVR-FAS, making it prone to aortic tissue damage and migration. Finally, although the TAVR-CAS offered a larger opening area, its paravalvular leakage was higher. This may be attributed to a similar thrombogenicity potential characterizing both models. The computational framework emphasizes the significance of mechanobiology in FAS and underscores the requirement for tissue modelling at multiple scales.

## 1. Introduction

Fibro-calcific aortic valve disease (FCAVD) is characterized by the growth of calcium or the accumulation of fibrosis in the aortic valve (AV) tissues [1]. In a progressive state it causes thickening of the tissues and narrowing of the valve leading to fibrocalcific aortic stenosis (FAS) and valve replacement eventually. Calcific aortic stenosis (CAS) due to calcific aortic valve disease (CAVD) has been a burden leading to high mortality in the Western world during the last 30 years [2]. Current studies suggest that FCAVD may have sex-specific pathophysiology and clinical presentation differences. It has been observed that women tend to develop the disease with less severe calcification compared with men [3]. Specifically, despite the potentially lower progression of the disease, women develop similar pathological haemodynamic progression as men [4]. This discordance between aortic valve calcification and haemodynamic severity of aortic stenosis (AS) suggests that fibrosis might be a more significant contributor to valvular dysfunction in women compared with men, and to the progression of AS [5].

Recently, the interplay between the structural and haemodynamic responses of CAVD has been extensively studied utilizing fluid–structure interaction (FSI) analysis [6]. Yet, none of these works investigated FAS cases. As the fibrotic tissues cannot be visualized by the current image modalities [5], *in silico* analysis can shed light on their biomechanical behaviours [7]. Calcified leaflets change the valve's haemodynamics dramatically. Weinberg & Kaazempur Mofrad [8] employed a multi-scale FSI model for tricuspid (TAV) and bicuspid (BAV) aortic valves. They computed the strain magnitudes at the cell level, where calcification initiation occurs, alongside the aortic side of the leaflet. In their subsequent study [9], they studied the ageing of the aortic valve using FSI model including tissue flexibility, degradation, thickening and calcification. Calcification was modelled by adding calcification nodules and by growing them for older ages. Calcification nodules are simulated by stiffening two-dimensional shell elements of the aortic valve. Van Loon [10] used an FSI model to study aortic stenosis (AS). The calcification was modelled by stiffening the leaflet material in two patterns: increased stiffness toward the valve's ring and increased stiffness toward the leaflet's free edge; in addition, by using constitutive models that vary by region, assuming that calcification progresses from the medial to the free end [11]. Analogously, Amindari *et al*. [12] simulated CAVD leaflet deformations and blood flow dynamics using FSI modelling. The calcified leaflets were modelled stiffer with a higher Young's modulus. Moreover, Javad *et al*. partially reduced the aortic valve cross-section area but did not model the calcification deposits. They evaluated the haemodynamics parameters in relaxation and exercise conditions [13]. Katayama *et al*. [14] examined the flow and stresses/strains of BAV and TAV aortic valves for stenotic cases where calcification was modelled by thickening the leaflet based on strain curvature. Chandra *et al*. [15] developed a two-dimensional FSI analysis of BAV and TAV including CAVD simulation. They assessed and compared the wall shear stress (WSS) of these two configurations. The calcification was modelled by stiffening the elements at the base of the leaflet for two kinds of calcification severity, mild and moderate. Two biomechanical parameters are known to affect the calcification process—tissue strain and haemodynamic shear loads [16–18]. It was suggested that local strain enhancement caused by a rigid core in the tissue can significantly impact the calcification growth [19]. In more advanced simulations, multi-layered calcified BAV models structure were constructed exploring the mechanism that drives CAVD progression in BAV patients. These results were compared with an *in vitro* pulse duplicator test [11]. In a recent study conducted by our group, we employed final elements analysis where we gained a deeper understanding of the structural characteristics associated with the simultaneous processes of tissue thickening and calcium growth. Specifically, we focused on female patients [7].

Currently, the most common therapy for aortic stenoses is aortic valve replacement surgically or via transcatheter procedures (transcatheter aortic valve replacement; TAVR) [13]. Two common complications, paravalvular leakage (PVL) and device thrombogenicity, are highly associated with TAVR post-procedure outcomes. Different studies have attempted to measure and validate PVL and its pathways, for different calcium disease morphologies. Three-dimensional printing techniques were used to assess the flow patterns and haemodynamics of PVL post-TAVR severity and were evaluated using computational fluid dynamics (CFD), and verified by utilizing four-dimensional flow magnetic resonance imaging (MRI) [20] or patient-specific *in vitro* replicas [21]. No correlation was found between the implantation depth and the extent of PVL [22]. In previous studies, we have investigated PVL for self- and balloon-expandable devices in BAV patients, including anchoring forces [23] and thrombogenicity potential calculations [24]. When considering the impact of sex on outcomes following TAVR procedures, the variation in the calcification-to-fibrosis ratio may contribute to the relatively better results observed in women as compared with men [25,26], as higher levels of calcification were linked to an increased risk of paravalvular regurgitation [27,28]. Other studies tried to evaluate TAVR device thrombogenicity potential making use of Lagrangian approach calculation of stress accumulation along individual platelet trajectories in the flow field for predicting blood damage and risk [29] including several seeding patterns, stochastic walk model, discrete phase method and simplified trajectory calculations with pathlines were carried [21,24,29–32]. Also, post-processing implementation options were evaluated including single and repeated passages stress accumulation (SA) with time averaging [33]. Studies also focused on the blood biochemistry and blood contact with foreign surfaces, with regards to micro-particle levels post-implantation [34]. Flow stasis showed a strong correlation with the thrombus volume in the neosinus [35]. Furthermore, the implantation depth and the device mechanism, self- or balloon-expandable, are directly related to the thrombogenicity potential in the main and bulk flow [36,37].

There is a paucity in the literature of numerical models assessing the risk of leaflet thrombosis post-TAVR in FCAVD patients, as well as FAS FSI models, e.g. such that include fibre remoulding with low calcium volume on AS. This combination is distinctive to female patients, leading to severe AS even with low calcium volumes compared with men. This study aims to evaluate the specific structural and haemodynamic factors of these two different mechanisms of AS and to predict their post-TAVR outcomes. A high calcium volume CAVD with no evidence of fibrosis, and collagen fibres remodelling combined with low calcium volume cases were investigated. Stenotic haemodynamics FSI factors, current AS severity according to clinical diagnostics and its post-TAVR outcomes, including paravalvular leakage and thrombogenicity footprint in self-expandable devices were studied. Given the fast-growing use of TAVR for various CAVD patients, it is essential to have a deeper understanding of the fundamental mechanisms of particular calcium or fibrosis disease morphologies and the role of gender in treating these patients.

## 2. Methods

### 2.1. Clinical data acquisition and population

**Figure 1.**
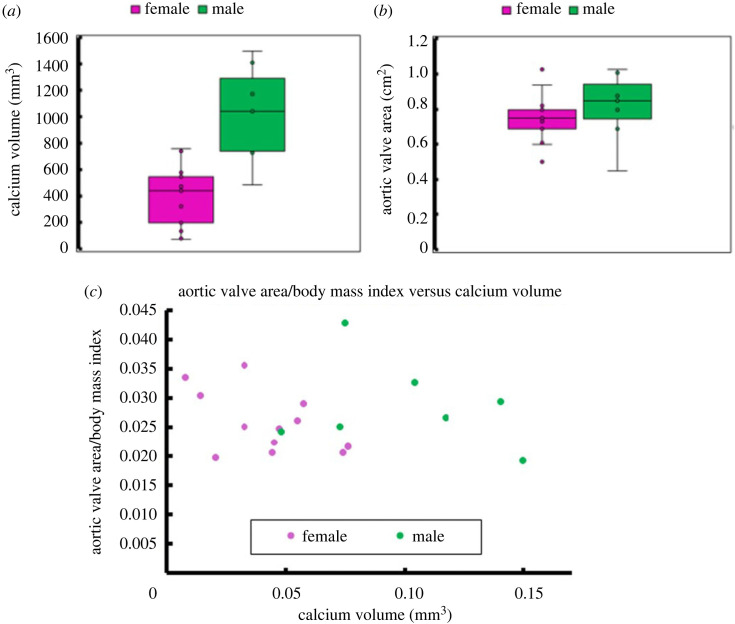
Sex-specific clinical data comparison between males (in green) and females (in pink): (*a*) Calcium volume median and deviation: 447 ± 353 mm^3^ versus 1043 ± 500 mm^3^. (*b*) Stenotic aortic valve area median and deviation: 0.75 ± 0.15 cm^2^ versus 0.85 ± 0.25 cm^2^. (*c*) Calcium volume effect on aortic valve area normalized by body mass index.

Computed tomography (CT) scans of pre-TAVR tricuspid aortic valve patients diagnosed with severe aortic stenosis were obtained from Rabin Medical Center, Israel. Data were anonymized and collected after approval from the hospital institutional review board committees (RMC 0636 approval) adhering to the Declaration of Helsinki. Following a protocol from our previous study [38], contrast-enhanced and electrocardiography-gated CT scans were acquired using a 256-channel volume CT scanner (Brilliance iCT, Philips Healthcare, Cleveland, OH, USA) with an in-plane resolution of 0.67 × 0.67 mm and slice thickness of 0.67 mm. At the diastolic phase, data from 19 severe aortic stenosis patients, 7 males and 12 females, was extracted from retrospective electrocardiogram (ECG)-gated cardiac CT scans, on 75% of the R-R interval. The data were normalized by the body mass index (BMI) of each patient to minimize the physiological dimensions' differences between the sexes (figure 1). Two female patients from this database were identified to have severe fibrocalcific aortic stenosis (FAS) with a combination of low calcium and fibrosis of the valve's tissue. One female representative case was chosen for the numerical analysis demonstrating the FAS effect and compared with CAS female case.

### 2.2. Proposed computational structural models for CAS and FAS valves

**Figure 2.**
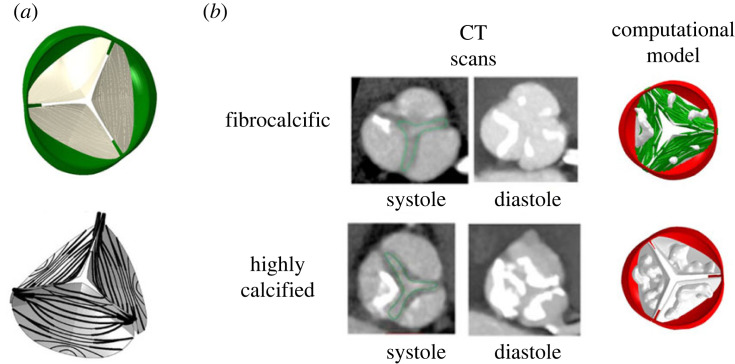
(*a*) Computational model of the parametric healthy tricuspid aortic valve with the collagen fibre network distribution. (*b*) Highly calcified and fibrocalcific aortic stenosis diseases computational models, including calcium deposits and fibres' architecture, reconstructed from their patients’ specific CT scans.

To ensure reliable finite-element (FE) computations it is imperative to achieve accurate geometric reconstruction of AV models. The current reconstruction method is based on a parametric representation of the aortic valve (figure 2*a*). The parametric model is used to represent different geometric dimensions of the aortic valve with its heterogeneous structure. The parametric aortic valve model that was developed in our group introduces a native three-dimensional geometry represented by selected parametric curves allowing for a general reconstruction and representation of its structure. From a CT scan of a specific patient, the geometric parameters are measured. By using mathematical curves, geometrical surfaces are reconstructed and a computational finite-element mesh is created [39]. The model takes into account the heterogeneous structure of the leaflets. In this study, the calibration of the fibres' architecture and volume was based on a sensitivity study examining the architecture types (type 1 and 2) and fibres' volume in structural finite-element simulations of different FAS models [7]. The calibrated values were set to match the aortic valve opening area, which is clinically measured. Furthermore, to model the CAS cases, the calcium deposits were embedded inside the leaflets which have variable thickness. This process resulted in calcification deposits located in confined ‘pockets’ within the soft tissue. The tissue and the calcium were sharing nodes in the boundary mesh between them, assuming full-interface displacement continuity. That said, the fibres and the calcium are embedded inside the leaflets (matrix) and share a mutual interface and hence an alternative interaction during the cardiac cycle subject to the different pressure loads [11,23]. For this study, we extend the average parametric healthy TAV and BAV geometric models to integrate calcific and fibrocalcific aortic stenosis pathologies. A representative case of fibrocalcific aortic stenosis was chosen from the pre-TAVR database with a 0.82 cm^2^ opening area and a measured maximum peak pressure gradient of 74 mmHg from Echo. A second case with high-volume calcific aortic stenosis, and a 0.73 cm^2^ opening area with a measured maximum peak pressure gradient of 99 mmHg, was investigated (figure 2*b*). Both cases present symptomatic severe AS according to American College of Cardiology/American Heart Association (ACC/AHA) guidelines [40]. The FAS parametric model includes a thickening collagen fibre network (CFN)-based leaflet tissue with small calcification volumes. The CAS model employs healthy CFN with embedded high calcification volumes based on a representative CT taken from a pre-TAVR patient [23].

**Figure 3.**
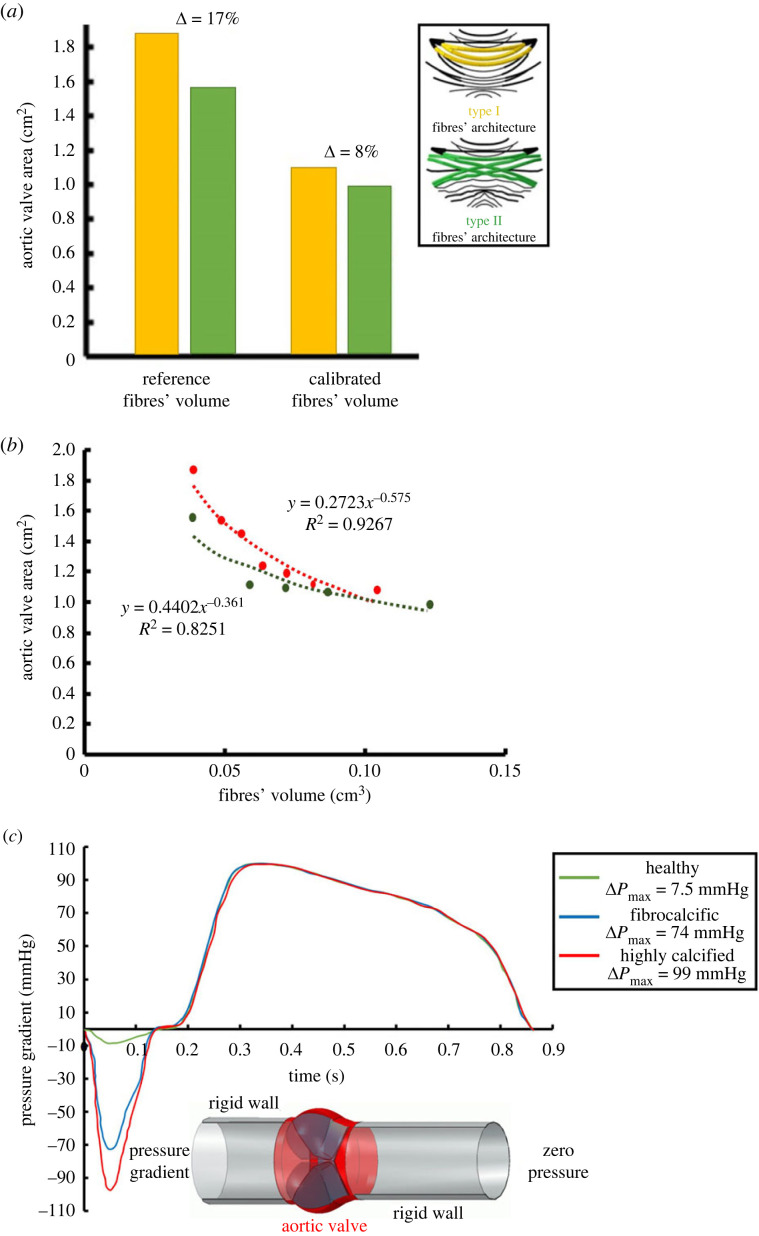
(*a*) Calibrated aortic valve area of the different fibres' architecture types (I in yellow and II in green). (*b*) Aortic valve area variation due to fibres’ volume for the different architecture types. (*c*) Fluid–structure interaction boundary condition of time-dependent pressure gradient for healthy, highly calcified and fibrocalcific models.

For the FAS model, the fibres' architecture and volume were calibrated to represent the CT-measured opening area of the current stenotic case. Examining the opening responses from finite-element structural analyses, it was found that the model with architecture-II fibres, having a threefold increased radius (varying from 0.01 to 0.04 cm [35]), yields a similar stenotic opening area. The computation used a patient-specific physiological pressure (figure 3*a*) as an imposed boundary condition and was compared with a healthy configuration [7]. Furthermore, it was found that increasing the fibres’ volume beyond threefold does not significantly reduce the opening area (figure 3*b*). The CFN fibres, calcium and leaflets were modelled using beam and three-dimensional solid wedge elements, respectively. In both CAS and FAS models, linear material properties were used for the CFN, leaflets and calcium deposits; 100 MPa, 1 MPa and 1 GPa, respectively. A density of 1100 kg m^3^ was used for the leaflets and the fibres with a Poisson ratio of 0.45. A mesh refinement study of the finite-element structure model was conducted based on our previous studies [11,41].

### 2.3. Fluid–structure interaction: staggered lattice-Boltzmann CFD and finite-elements

The haemodynamic fluid–structure interaction computational models of the highly calcified and fibrocalcific aortic stenosis cases were performed using an integrated lattice Boltzmann method–finite element (LBM-FE) refined modelling methodology to investigate the complete haemodynamics responses for the entire cardiac cycle. To that end, we extend our recent LBM-FE fluid–structure interaction methodology for healthy and congenital AVs [41] to represent pathological cases of highly calcified and fibrocalcific aortic stenosis. The Boltzmann equation describing the statistical behaviour of population of particles under elastic collisions among them was used for simulating fluid in this study. The fluid density and momentum are retrieved by averaging the decomposition of each infinitesimal volume of fluid into a population of particles. The particles' population is discretized into a reduced set of discrete velocities determining their likelihood of motion in a given direction. A multi-relaxation time collision operator was employed according to central momentum space in a three-dimensional Cartesian velocity set using a D3Q27 uniform lattice. The flow was assumed to be laminar and the blood to be Newtonian. The XFlow (Dassault Systemes, Simulia Corp.) commercial code was employed to simulate the CFD of native FAS-CAS aortic valves pre- and post-TAVR. A grid refinement study of the fluid domain was conducted in the FSI simulations (see [41]). Two cylinders with a length equivalent to three times the valve's diameter were added in the upstream and downstream, where a pressure gradient boundary condition was applied to eliminate the effect of boundary conditions on the region of interest (the valve's leaflets). The downstream pressure outlet was set to zero, and a time-dependent pressure gradient was applied upstream. For each of the four cases: TAV, BAV, CAS and FAS, a tailored pressure amplitude was employed (figure 3*c*). The amplitude values were calibrated based on the Echo report of each patient, corresponding to their peak pressure gradient through the cardiac cycle. The peak pressure gradient of the pathological models was taken from the Echo report of each case, while the rest of the waveform was calibrated according to the healthy case. This allows a representative calibration of the complete waveform of each patient, taking into account its corresponding peak pressure gradient that controls its structural and haemodynamic responses. A two-way coupling partitioned approach was used to solve the FSI for both simulations, where XFlow and Abaqus solvers (Dassault Systemes, Simulia Corp., Providence, RI) were used for fluid and structural parts, respectively. A co-simulation engine was used to manage communication between the solvers. An explicit flow solver was used for the LBM flow equations and transferred the traction load on the leaflets, which included fluid pressure and shear stress, to the structural solver. The implicit nonlinear structural solver determines the displacements by iteration until convergence and transfers the new geometry back to the flow solver. This iterative-staggered process was repeated for each step until the end of the simulation. The simulations were executed on a local workstation featuring an Intel(R) Core(TM) i9-10980XE CPU@ 3.00 GHz, with each solver allocated eight cores in turn. Both simulations required approximately 144 h to complete. A frictionless master-slave contact algorithm was applied between the leaflets in the structural part.

### 2.4. TAVR deployment procedure, paravalvular leakage and thrombogenic footprint prediction

The simulation involved implanting a self-expandable TAVR valve (Evolut PRO, Medtronic, Inc., Minneapolis, MN) stent with a diameter of 29 mm into the highly calcified and fibrocalcific aortic stenosis at a 4 mm depth, which was defined as the distance between the aortic annulus and the intraventricular end of the TAVR stent according to [42]. The stent was made of Nitinol (user material (VUMAT) including 14 constants available in Abaqus) as described in [43], and it was crimped by applying a radial boundary condition on the nodes of a cylinder until it fit into a 36 mm diameter catheter. The leaflets were opened to create enough space for the catheter equipped with the crimped stent to be positioned inside the native aortic leaflets. Finally, the stent was deployed by pulling the catheter axially, which allowed for a gradual expansion of the stent. During the crimping procedure, a frictionless contact was defined between the cylinder, the stent's struts and the aortic leaflets. During the deployment, the TAVR valve prosthetic leaflets and cuff were not included to reduce the large computational efforts [44]. The analysis was performed with a minimum time step of 10^−7^ using a semi-automatic mass scaling applied for the entire model. The FE-Abaqus Explicit solver was used to run the deployment simulation. The stent anchorage and the maximum contact pressure were calculated. The stent anchorage forces were evaluated based on the contact area between the stent and the native leaflets. Summation of the surface area of all the elements in contact with the stent yielded the total contact area. Maximum contact pressure was calculated using the maximum contact force value divided by the area of the corresponding element [23].

To determine the potential for paravalvular leakage and thrombosis in highly calcified and fibrocalcific aortic stenosis, a separate CFD simulation was conducted on the deployed TAVR. The final and full TAVR valve model is accomplished by attaching the prosthetic leaflets and a cuff to the deployed stent frame. The latter configurations are determined using a specified relative displacement field from the original pre-crimped reference configuration. A structural analysis procedure is used for the last step using the displacement field as a boundary condition. The prosthetic leaflets and cuff, made of the glutaraldehyde-treated porcine pericardium, were modelled using an Ogden third-degree isotropic hyperelastic material model based on properties obtained from [45]. The prosthetic leaflets were in contact with each other and the stent through Coulomb friction. The domain created by the TAVR geometry is used for both CFD-FSI analyses and also utilized for the paravalvular leakage calculations (figure 3*c*). CFD analyses with a constant pressure of 90 and 0 mmHg were employed in the aortic and left ventricle extensions, respectively, representing an average diastolic pressure gradient. A graded grid refinement was resolved to save computational time, where the fluid domain is composed of different lattice sizes and a fixed time step of 0.01 ms. The grid was reconstructed from a uniform 1 mm lattice resolution on the far field, where two refinement levels towards the region of interest (the valve) were determined. This allows an acceptable resolution of 0.25 mm in calculating the paravalvular leakage routes via the small gaps and saves computational time in the far field. A temporal convergence and grid independence studies of different time steps and lattice resolutions were conducted to ensure that the chosen grid and time step would not affect the paravalvular leakage values. To quantify the paravalvular leakage for each case, the flow volume in the plane located below the annulus was calculated by integral summation of the longitudinal velocity component for a period of 500 ms representing the diastolic phase.

Next, a thrombogenic footprint approach, estimating the SA probability density function (PDF) of seeded particles, was utilized [33,36,37]. The probability density function represents the statistical distribution of the stress accumulation values achieved by each platelet at the endpoint of its individual flow trajectory. To guarantee that the percentage activation is independent of number of seeded particles and spatio-temporal variations, we use bootstrapping statistics to interpolate between smaller and larger populations. This approach guarantees that the statistical distributions extracted from different population sizes are compatible and comparable. Besides solving the transport equations for the continuous phase, the flow solver also calculates the transport of a discrete phase consisting of spherical particles dispersed in the continuous phase. Therefore, using this Lagrangian formulation that includes the discrete phase inertia, it is possible to evaluate particles' trajectory, hydrodynamic drag and the external forces’ effect on the steady flow. Given each single particle velocity spatial derivatives and locations at each time increment, their shear stress, assuming laminar flow, is calculated, using an in-house Matlab code, by

2.1 $$\tau_{ij}\approx \mu \left(\frac{\partial u_{i}}{\partial x_{j}}+\frac{\partial u_{j}}{\partial x_{i}}\right)\;,$$

where μ is the viscosity and $\partial u_{i}/\partial x_{j}$ is the velocity gradient. Next, the instantaneous stress metric σ is calculated, for each particle, from the $\tau_{ij}$ components according to

2.2 $$\sigma =\frac{1}{\sqrt{3}}\sqrt{\tau_{11}^{2}+\tau_{22}^{2}+\tau_{33}^{2}-\tau_{11}\tau_{22}-\tau_{22}\tau_{33}-\tau_{11}\tau_{33}+3(\tau_{12}^{2}+\tau_{23}^{2}+\tau_{13}^{2})},$$

and finally, the SA, which is the summation of the instantaneous stress magnitude and exposure time product during the particle's trajectory duration $\left(T_{\mathrm{trajectory}}\right)$, is approximated via

2.3 $$\mathrm{SA}=\;\int_{0}^{T_{\mathrm{trajectory}}}\sigma dt\approx \sum \sigma \cdot \Delta t\;,$$

where the SA is directly used for the PDF generation.

Thus, to assess the thrombogenic footprint PDF of the implanted device for the calcific and fibrocalcific aortic stenosis cases, 10 000 uniformly distributed particles were injected with an initial longitudinal velocity of 1 m s^–1^ in the CFD-solved fluid domain near the aortic side. A dedicated in-house code was used for this post-processing procedure. These particles have physical properties such as a density of 1125 kg m^−3^ and a diameter of 3 µm, assuming pure elastic wall collisions with no particle-to-particle collisions. A convergence study was conducted to ensure that the selected number of particles would not affect the sensitivity of the PDF.

## 3. Results

### 3.1. Sex-specific disease development

The median calcium volume and aortic valve area (AVA) were 447 ± 353 mm^3^ and 0.75 ± 0.15 cm^2^ for the female group and 1043 ± 500 mm^3^ and 0.85 ± 0.25 cm^2^ for the male group, respectively (figure 1). It is evident that female patients with lower calcium volume than males develop the same AS severity. Our hypothesis in this study is that female patients present more fibrosis and denser connective tissue than males for the same AS degree [5]. The CT scan in figure 2*b* allows for the observation of the extent of fibrotic tissue in the area, where FE simulations were conducted to calibrate the fibre architecture shape and volume demonstrating its effect on the leaflets opening.

### 3.2. Flow field responses

**Figure 4.**
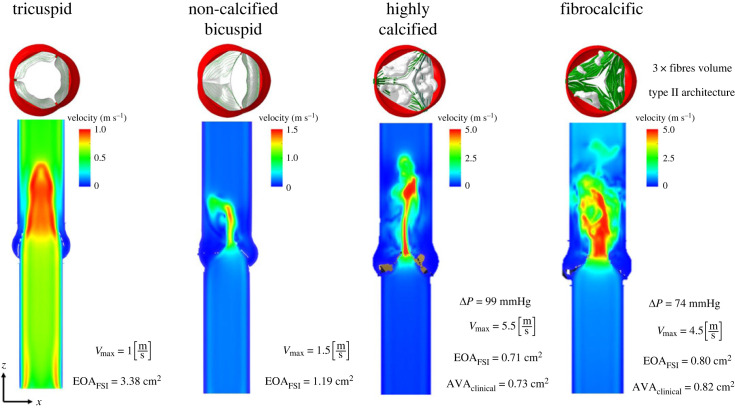
Fluid–structure interaction models using coupled LBM-FE: maximum velocity jet at systole peak and effective opening area for healthy tricuspid, non-calcified bicuspid, highly calcified and fibrocalcific aortic valves models.

Figure 4 presents various fluid–structure interaction analyses of four models of the aortic valve; healthy TAV, congenital BAV, CAS and FAS at systole peak. The TAV featured a centrally placed jet and achieved a maximum velocity of 1 m s^−1^, while the BAV had an eccentric jet and reached a maximum velocity of 1.5 m s^−1^. Their respective effective orifice areas (EOA) were 3.38 and 1.19 cm^2^ respectively. Both valves were subject to a healthy pressure gradient. Furthermore, for stenotic CAS and FAS the calculated EOA was 0.71 and 0.80 cm^2^, corresponding to clinically measured values of 0.73 and 0.82 cm^2^, respectively. Each case respective to its specific pressure gradient, developed a narrowed jet with very high velocity magnitudes (5.5 and 4.5 m s^–1^, respectively).

### 3.3. Mechanical and wall shear stress responses

**Figure 5.**
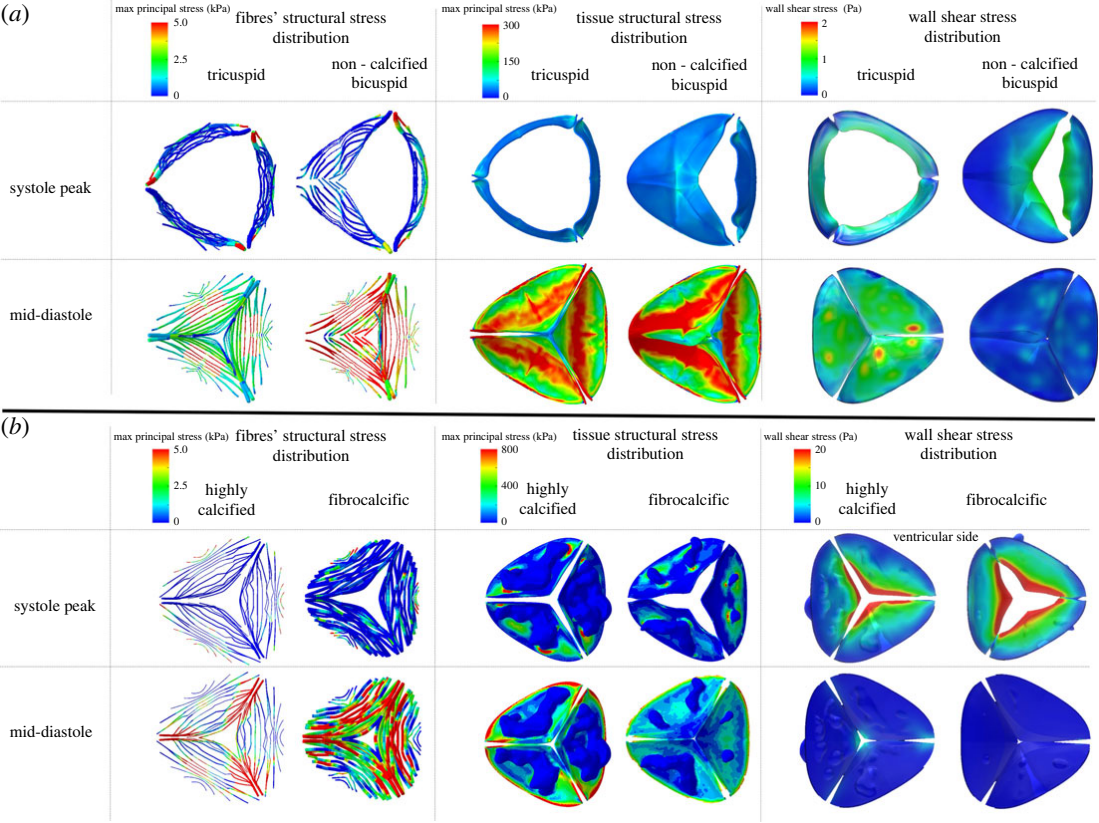
Fibres’, tissue and wall shear stress distributions at systole peak and mid-diastolic phase for (*a*) tricuspid, non-calcified bicuspid, (*b*) highly calcified and fibrocalcific models.

Figure 5 presents the mechanical stresses load within the leaflets and the CFN, as well as the wall shear stress (WSS) for CAS and FAS at systole peak and mid-diastolic phase. In both models, during the systole peak (figure 5*b,* first row, first column) the stresses in the fibres develop near the lower attachment line of the leaflets with the aortic root, whereas in the FAS case, the stress is also developed in the upper belly region. This happens in two FAS leaflets (the upper and right ones). Where in the tissue, the stresses are dispersed around the calcium deposits region (figure 5*b*, first row, second columns). At the mid-diastolic phase (figure 5*b*, second row, first columns), the stresses in the fibres develop near the commissures mainly and gradually radially decrease towards the belly and the bottom attachment line. However, in the FAS case, a high stress is also developed in the belly region towards the tip of two of its leaflets. During both the systole peak and mid-diastolic phases, the stresses distributions in the collagen fibre network in the FAS resemble those of the CAS case, despite being subjected to a lower pressure peak gradient. In both models, the stresses in the tissue are dispersed around the calcium deposits area (figure 5*b*, second column). Nevertheless, for the CAS the stresses are higher. It is noted that there is a mismatch between the developed stresses in the leaflets and the fibres. This can be interpreted due to the rigid calcium bulks that resist the aortic valve opening forces that lead to elevated stresses around them. In addition, the stresses in the fibres are two orders of magnitude lower than in the tissue for both models, and stress concentration areas near the edges of the deposits in the tissue are observed.

The WSS is presented on the ventricular side only (figure 5*b*, third column). It is expected that the unidirectional flow during the systole peak generates higher WSS than the recirculating flow on the aortic side. The WSS is approximately 20 MPa in the tip region and gradually decreases towards the belly region in both models. Nevertheless, in the fibrocalcific aortic stenosis case the WSS distribution near the tips of the leaflets is larger and tapers off more slowly than the highly calcified aortic stenosis. Furthermore, it is observed that the WSS is significantly higher at the systole peak, whereas it is negligible at the mid-diastolic phase. The observed phenomenon can be attributed to two factors: the high pressure gradient experienced during the peak of systole and the partially restricted opening of the leaflets. This restriction causes a hindrance to the backward vorticity during the closing phase, leading to negligible WSS values.

### 3.4. Post-TAVR procedure outcomes

**Figure 6.**
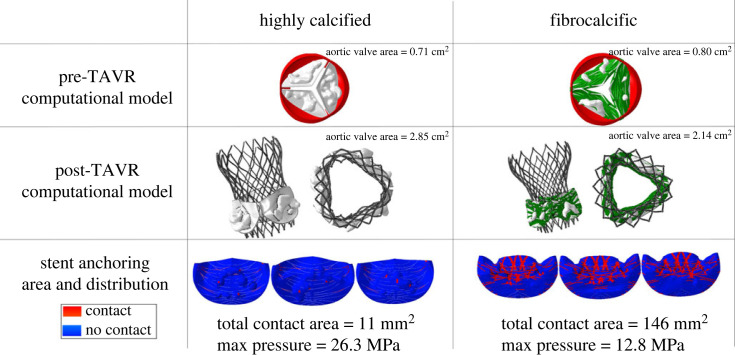
Pre-TAVR computational models and their post-TAVR aortic valve area, anchoring contact area at each leaflet and maximum deployed contact pressure of highly calcified and fibrocalcific structural models.

While TAVR restores blood flow, it is hampered by post-procedural complications that hinder its clinical outcomes. This was studied by examining and quantified the post-TAVR opening area, anchoring area and maximum contact pressure on the leaflets, PVL and thrombogenicity potential in the severe aortic stenosis highly calcified and fibrocalcific cases. The structural analysis revealed that the post-TAVR aortic valve area increased from 0.71 to 2.85 cm^2^ and 0.80 to 2.14 cm^2^, respectively (figure 6, rows 1 and 2). Side-by-side comparison showed that the anchoring area of FAS is significantly higher (146 versus 11 mm^2^), whereas in CAS the maximum applied contact pressure on the native leaflets is approximately doubled (26.3 versus 12.8 MPa). Moreover, the contact distribution in CAS is distinct to the calcium deposits surface, which prevents the deployed stent from contacting the leaflets directly. On the other hand, because of the low calcium volume and smaller deposits, the contact area in FAS surrounds a notable portion of the leaflets area (figure 6, row 3).

**Figure 7.**
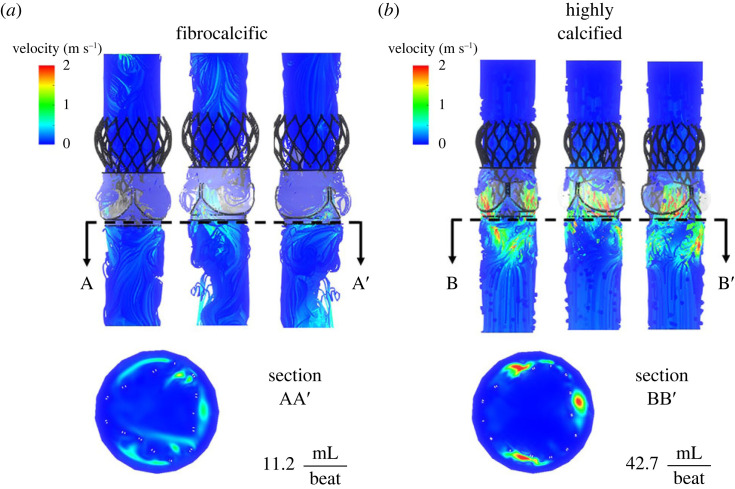
Velocity magnitude contours on different rotational views showing paravalvular leakage jets of highly calcified and fibrocalcific models at diastole peak time point. Paravalvular leakage quantification on sections AA’ and BB’ under the valve's annulus.

The haemodynamic analysis of post-procedure outcomes showed that the paravalvular leakage in CAS is higher than in FAS (42.7 versus 11.2 ml/beat) (figure 7). The mild and severe paravalvular leakage in CAS and FAS, respectively, is directly related to the anchoring of the stent. Furthermore, it is noticeable that in the FAS case (figure 7*a*), the PVL channels tend to be predominantly localized near the commissures of the aortic valve rather than in the gaps between the native leaflets and the stent—as in the case of CAS (figure 7*b*), where a high velocity leakage jet is observed.

**Figure 8.**
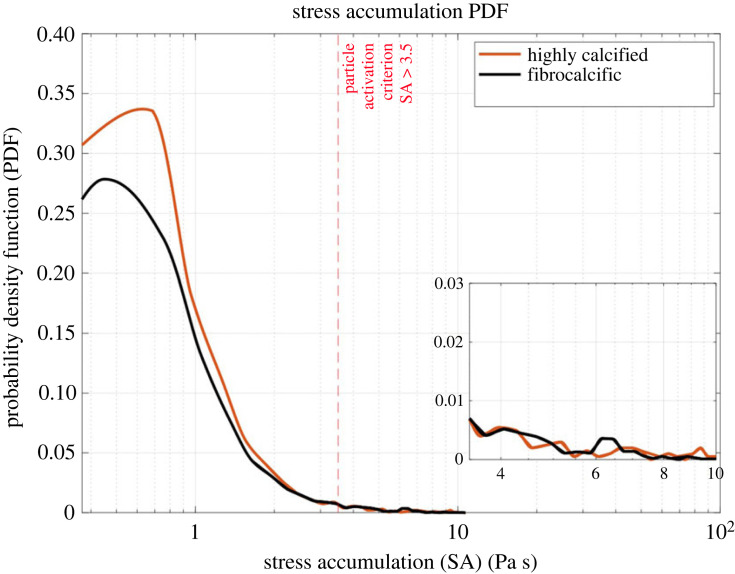
Stress accumulation probability density function of TAVR implantation in logarithmic scale for highly calcified and fibrocalcific models in bulk flow (main plot) and tail region (inset). The vertical red line is Hellum's criterion threshold for particle activation.

The device thrombogenic footprint was estimated in the different pathologies, where a criterion for platelet activation threshold was used based on Hellum's criterion [33] (figure 8). Following this criterion, particles that have SA value higher than 3.5 Pa s threshold are activated. The focus was on two regions; the main mode (bulk flow) and the tail region, which represents platelets exposed to higher SA values, hence higher activation potential. It was estimated that about 6.2% in CAS and 5.8% in FAS of the platelets are activated. Both diseases are probably characterized by similar thrombogenicity where the probability in CAS is significantly higher in the bulk flow yet incidental and intermittent in the tail region.

## 4. Discussion

We introduce predictive computational methodology for evaluating the flow patterns coupled with the structural responses in post-procedure TAVR outcomes in severe aortic stenosis. Detailed biomechanical analysis of aortic stenosis, congenital BAV, CAS and FAS, indicated different responses that require tailored disease handling and management to be considered [46]. This study extended our previous models in congenital calcified and non-calcified BAV [11,41] to inspect TAV calcific and fibrocalcific cases. Specifically, LBM-FE fluid–structure interaction simulations of the common highly calcified and the notably fibrocalcific aortic stenosis case, which is strongly influenced by sex differences and is more prevalent in female patients, were explored. Additionally, post-TAVR deployment outcomes in respect to paravalvular leakage and device thrombogenic potential were investigated and compared using FE and CFD methods. To our knowledge, this is the first study to apply LBM-FE fluid–structure interaction simulations of a fibrocalcific aortic stenosis case. While our methodology is designed to provide insights, its predictive capabilities and clinical applicability are contingent upon thorough validation.

Considering the structural and haemodynamic aspects, it is evident that the mechanical and WSS distributions are complex, as anticipated due to the irregular and heterogeneous nature of aortic valve pathologies. The interaction between calcium deposits and the surrounding soft tissue, as well as the fibre architecture, plays a crucial role in the FSI simulations. It directly impacts both the haemodynamics and structural responses. Figure 5 shows that the stresses are higher in the soft tissue and fibres than in the calcium deposits for both highly calcified and fibrocalcific aortic stenosis [47]. This is because the tissue and fibres are softer and more deformable than the calcium deposits, so they will undergo more deformation and experience higher stresses in response to the applied load. Because of a significant difference in stiffness of the materials, local stress concentrations are created and compounded stresses transformed in the different regions of the leaflets. The calcium deposits that are embedded in the soft tissue generate distinct localized regions of elevated stress concentrations that increase the calcium burden and may lead to its rupture. In CAS case, at the systole peak the stresses are mainly concentrated near the commissures, sustained by the fibres and the tissue only, as the calcium deposits cover the majority of the leaflets' surfaces. Similarly, at the mid-diastolic phase, the stresses are concentrated also near the coaptation area and in the fibres near the commissures. In FAS, the same trend is observed, yet additional elevated stresses are developed in the fibres—specifically between the belly and the tips of the leaflets (the fibrotic area). The stresses imposed on the leaflets not only contribute to the resistance of their opening and closing, but also exacerbate the severity of aortic stenosis, in addition to the resistance caused by calcium deposits. Moreover, in both cases, regional stress concentration areas near the calcium deposits edges are developed in the tissue. The peak stress values were also found in the vicinity of the calcification. Thus, in FAS, the fibres contribute to increased stiffness in regions that lack calcium deposits, influencing the overall stiffness of the leaflet. This demonstrates the dominance of the fibres in the FAS disease stenosis. Consequently, the presence of calcification and fibrosis may hasten the progression of AS by creating a mismatch in compliance and subsequent concentration of stresses along its perimeter [48]. The stiffer behaviour of the combined fibrous and calcified leaflets composition explains the higher jet velocity magnitudes at systole peak in CAS and FAS (figure 4). Accordingly, WSS is higher and localized as compared with the non-calcified tricuspid and bicuspid AVs, as we previously described [11,41]. This is expected, as CAS and FAS are subject to higher pressure gradients, limited EOA and narrower systolic jets (figures 4 and 5).

Post-TAVR performance metrics measurements indicate that those are mostly morphology-dependent [49]. Hence, we examined TAVR post-procedure outcomes in two models of the leading causes of aortic stenosis: CAS with high calcification, and FAS with small volume calcium load combined with fibrosis. The CAS has a higher maximum contact pressure and smaller contact area values than FAS (figure 6), making it more prone to aortic tissue damage and migration or dislodgement. The stent's anchoring forces on the leaflets in FAS are higher, yet the opening area after TAVR is triangular and smaller than in CAS, whose area is circular and larger (figure 6). This emphasizes the importance of the fibres' architecture and location in resisting the TAVR deployment as compared with the bulk deposits in CAS. The bulk calcium deposits detach the stent from the native leaflets, consequently reducing its anchoring. However, type II fibre architecture in FAS prevents the stent expansion in the denser fibres vicinity while allowing its expansion elsewhere, resulting in triangular opening. Moreover, the valve leaflets in FAS are thicker and more fibrous, making it more challenging to position and deploy the TAVR properly. To optimize the stent's anchoring, a different implantation depth could be considered [37] or alternatively a surgical valve's replacement.

The CFD analysis shows that the PVL is stronger in highly calcified aortic stenosis than fibrocalcific aortic stenosis (figure 7). Both models are characterized by multiple regurgitant jets, yet in calcific aortic stenosis, the jets formed in between the gaps of the native leaflets and TAVR with high velocity magnitude, whereas in fibrocalcific aortic stenosis, the jets formed near the commissures with relatively low-velocity magnitudes (figure 7). This was probably caused by the different opening morphology after TAVR. Furthermore, both models have a similar thrombogenicity potential PDF (figure 8). It was shown that TAVR in highly calcified aortic stenosis develops a marginally higher thrombogenicity potential than fibrocalcific aortic stenosis in the bulk flow, yet it is incidental and intermittent in the tail region. The tail region indicates the probability of platelets with a higher potential for activation. Thrombogenicity and the degree of PVL are intimately related in TAVR [21]. Furthermore, while in the bulk flow there is only a marginal difference in the thrombogenic potential between the fibrocalcific and highly calcified cases, in the tail region (figure 8, tail region: inset) the fibrocalcific case has a clear peak and most of the PDF curve is above the highly calcified case—in that SA range that is considered the far riskier range for thrombogenicity. While this may appear less prominent, it represents a large number of platelets that will be pushed beyond their activation threshold. Those will recruit other quiescent platelets that will aggregate and further boost the coagulation cascade. Even small differences in the number of activated platelets manifest into significant differences in the thrombogenicity.

### 4.1. Limitations

Certain limitations are acknowledged when interpreting the findings of this study. The accurate identification of fibrosis geometry from CT scans is best achieved by experts. The presence of a grey area in the leaflets can be interpreted as fibrotic tissue, which may have additional functional consequences, as discussed in [50]. The fibres architecture was idealized and calibrated to fit the opening area measured from the CT scan. However, a high-resolution MRI or scanning electron microscopy (SEM) is needed for histology analyses to determine them more accurately. The calcium deposits identified from the CT scans were scaled to adapt to the average parametric AV model, allowing for better comparison between the different models. In addition, it should be noted that the material properties of the various aortic valve elements were assumed to exhibit linear elasticity [11], which may impact the accuracy of stress predictions. Consequently, the stress values cannot be independently verified and are probably influenced by the validated metrics of the flow field and effective orifice area. The calcified leaflets were assumed at a stress-free state that may underestimate the maximum stress predicted by the model.

Our objective was to conduct fluid–structure interaction simulations for an entire cardiac cycle, striking a balance between the structural and fluid grids, with the LBM model assuming laminar flow. However, the flow regime in such valvular flow is laminar-transitional in certain phases of the cardiac cycle, with a possibility of turbulent behaviour occurring [41] that requires a finer grid to capture. Our laminar flow assumption is rooted in the comparative nature of our study; combined with the complexity of our FSI approach that involves structural intricacies of both calcific and fibrocalcific leaflet behaviour we find the laminar assumption to be adequate. We do acknowledge that turbulent stresses may contribute to stress accumulation on the platelets that might differ for different valves and pathologies. Yet again, it is a reasonable assumption for comparative studies such as ours. In future studies we plan to combine a transient turbulence model in our complex FSI model. As the TAVR outcomes are tightly coupled to the complex interaction between the patient's AV and the anatomy of the surrounding diseased tissue, those need to be considered on a case-by-case basis. The effect of different TAVR sizes, different implantation depths and device types (balloon- or self-expandable) should be considered in optimizing the different outcomes for each patient [22,51]. In this study, our objective was to conduct a comparative analysis rather than proposing a specific procedure or optimized outcomes. Finally, the limited sample size of only two patients' models without post-TAVR CT scans is acknowledged. However, these two cases offer for the first time a comparative analysis of CAS and FAS. This study serves as a methodological approach demonstrating that the thrombogenic potential and PVL quantification can be obtained in these models. This should be further validated in the future with *in vitro* platelet activation experiments of these models’ thrombogenicity footprint.

## 5. Conclusion

This study investigates calcific aortic valve haemodynamics that incorporate biomechanical parametric models of highly calcified aortic stenosis and fibrocalcific aortic stenosis patients undergoing transcatheter aortic valve replacement. For the first time, this study presents a computational framework that incorporates fluid–structure interaction for the retrospective clinical case of FAS. The models offer quantitative information on the flow field, structural and flow stresses, TAVR anchorage, paravalvular leakage, and their thrombogenic potential. We found that the interaction between calcium bulks with the surrounding soft tissue and fibres significantly affects the haemodynamics and structural responses, including regional stress concentrations, specifically demonstrating the dominance of fibres and their architecture in such stenotic valve. We additionally examined TAVR post-procedure outcomes and found that CAS has a higher maximum contact pressure and lower contact area values than FAS, which may make it more prone to aortic tissue damage and migration or dislodgement. The opening area after TAVR in FAS was triangular and smaller than in CAS. This further highlights the effect of the fibres' architecture and location in resisting the TAVR deployment as compared with the bulk deposits that are dominant in CAS. Finally, although it is usually assumed that CAS and FAS share a similar thrombogenic potential, we predicted that the paravalvular leakage is higher in CAS. It is evident from this study that different pathologies of aortic stenosis exhibit distinct behaviours in patients and should therefore be diagnosed and treated on an individual basis. Specifically, it highlights the need for further studies of FAS disease and progression for better diagnosis and treatment. Future studies with a larger group of patients are needed to confirm the predictions of the models. The findings of this study highlight the potential of biomechanically based numerical models as valuable tools for predicting the behaviour and mechanisms of different pathologies in aortic stenosis and hold promise in predicting post-operative TAVR outcomes for those patients.

## Data Availability

The datasets supporting this article have been uploaded as part of the electronic supplementary material [52].
